# Horns, nails, and leaky kidneys: A rare case of congenital nephrotic syndrome 

**DOI:** 10.5414/CNCS111949

**Published:** 2026-02-22

**Authors:** Dwarak Sampath Kumar, Mythri Shankar, C G Sreedhara

**Affiliations:** Department of Nephrology, Institute of Nephro-Urology, Bengaluru, Karnataka, India

**Keywords:** nail-patella syndrome, nephrotic syndrome, focal segmental glomerulosclerosis, LMX1B mutation, genetic kidney disease

## Abstract

Nail-patella syndrome (NPS) is an uncommon autosomal dominant condition marked by nail dysplasia, skeletal abnormalities, and variable renal manifestations, resulting from mutations in the *LMX1B* gene. We report a rare case of a 23-year-old male presenting with nephrotic-range proteinuria, characteristic skeletal manifestations of NPS, and a family history of renal failure. Genetic testing identified a previously unreported heterozygous missense variant in the homeodomain of *LMX1B* (c.791A>C; p.Gln264Pro), supporting its pathogenicity. The absence of patellar hypoplasia in our patient highlights the phenotypic variability of NPS. This case reinforces the importance of detailed physical examination and targeted genetic testing in diagnosing nephrotic syndromes.

## Introduction 

Nail-patella syndrome (NPS) (OMIM 161200), or hereditary osteo-onychodysplasia, is an uncommon autosomal dominant condition caused by mutations in the *LMX1B* gene on chromosome 9q34 [1, 2]. The estimated prevalence of NPS is around 1 case per 50,000 individuals [1]. It typically presents with nail dysplasia, patellar hypoplasia, elbow anomalies, and iliac horns. *LMX1B* encodes a LIM-homeodomain transcription factor that plays a vital role in dorsal limb patterning and in maintaining podocyte integrity [3, 4]. Its mutations can impair podocyte function, causing proteinuria and reduced estimated glomerular filtration rate in 10 – 40% of patients, and progression to end-stage kidney disease (ESKD) in ~ 5% [5, 6]. 

## Case report 

A 23-year-old male presented with 1 month of bilateral pedal edema. Examination revealed fixed elbow flexion deformities with antecubital webbing (Figure 1A), dystrophic thumbnails and toenails (Figure 1B), and crepitus in the knees. His mother had similar skeletal anomalies and died of renal failure at age 43 after 2 years of dialysis. 

He was the only child born to his parents. Laboratory parameters were suggestive of nephrotic syndrome (Table 1). Pelvic X-ray showed bilateral iliac horns (Figure 1C). Knee X-rays showed normal patellae. Renal ultrasound showed normal-sized kidneys with mild cortical echogenicity. Renal biopsy revealed focal segmental glomerulosclerosis (FSGS) (not otherwise specified (NOS) variant) with 20% interstitial fibrosis. Immunofluorescence showed no significant staining for IgG, IgA, IgM, C3, or C1q. Electron microscopy was not performed due to financial constraints (Figure 1D). 

## Genetic testing 

Whole exome sequencing identified a heterozygous missense variant in exon 5 of the *LMX1B* gene (c.791A>C; p.Gln264Pro). The variant lies within the homeodomain region, which has been shown to be damaging in multiple in-silico tools, but it was classified as a variant of uncertain significance. However, in the clinical context, it supported the diagnosis of NPS. 

## Discussion 

NPS is an uncommon inherited condition passed down in an autosomal dominant manner, primarily marked by abnormalities in the nails and skeleton, and occasionally affecting kidney function in some individuals. Among the extrarenal features, iliac horns [3] are considered pathognomonic, while nail dystrophy – especially of the thumbs – is seen in nearly all cases (Table 2). Despite their high prevalence, patellar and elbow anomalies may be variable, and their absence does not exclude the diagnosis [4]. 

Kidney involvement remains the primary determinant of long-term morbidity and mortality [6]. Approximately 10 – 40% of individuals develop kidney manifestations, most commonly proteinuria of varying severity [7] (Table 3). A smaller percentage progresses to ESKD. The most frequently reported histopathological lesion is FSGS, often associated with varying degrees of interstitial fibrosis. In our case, renal biopsy revealed FSGS (NOS variant) with 20% interstitial fibrosis, consistent with the expected histology in NPS-related nephropathy. 

The condition is caused by mutations in the *LMX1B* gene, located on chromosome 9q34. This gene produces a LIM-homeodomain transcription factor that plays a key role in shaping the dorsal side of limbs and is vital for the development and proper function of podocytes in the kidneys [7]. It regulates key podocyte-associated proteins, including CD2AP and NPHS2 (podocin), and type IV collagen subunits in the glomerular basement membrane (GBM). Murine models have demonstrated that loss-of-function mutations in the gene *LMX1B* causes decreased GBM collagen expression and disruption of podocyte integrity [8, 9]. 

Genetic analysis through whole exome sequencing identified a single-copy missense mutation (c.791A>C; p.Gln264Pro) in exon 5 of the *LMX1B* gene, affecting the homeodomain region of the protein. While this variant has not been previously documented and is currently classified as having uncertain significance, several computational tools predict it to be harmful. When interpreted in the context of the patient’s characteristic skeletal abnormalities and strong family history of renal failure, this variant is highly suggestive of pathogenicity. 

Management of renal disease in NPS is largely supportive. Immunosuppressive therapy is generally not effective, as the disease mechanism is not immune-mediated. Angiotensin receptor blockers and diuretics form the mainstay of treatment to control proteinuria and edema. Our patient was treated accordingly and was counselled for kidney transplantation. Electron microscopy could not be performed due to financial constraints, but it may have provided further diagnostic insights by revealing characteristic GBM changes [10]. 

## Conclusion 

This case highlights a rare presentation of NPS with nephrotic-range proteinuria confirmed by genetic testing revealing a novel LMX1B homeodomain variant (p.Gln264Pro). The absence of patellar hypoplasia underscores the variable phenotypic expression of NPS. Early recognition through physical examination and radiographic findings helped avoid unnecessary immunosuppression. 

In young patients with nephrotic syndrome and skeletal anomalies, syndromic causes like NPS should be considered. Genetic testing can confirm the diagnosis, guide management, and enable family screening. Recognizing these clues can prevent misdiagnosis and facilitate appropriate, conservative treatment. 

## Authors’ contributions 

DSK collected the data and wrote the article. MS collected genetic data and wrote the article. SCG reviewed the article.

## Funding 

None. 

## Conflict of interest 

The authors have no conflicts of interest to declare. 

**Figure 1A:**
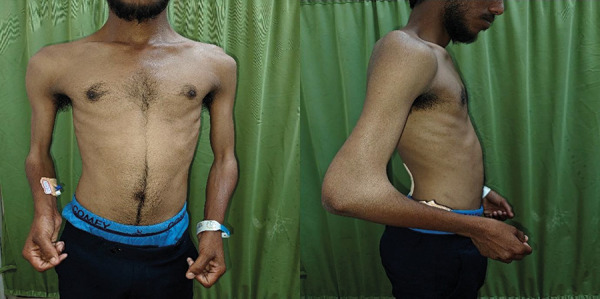
Fixed elbow flexion deformities with antecubital webbing.

**Figure 1B:**
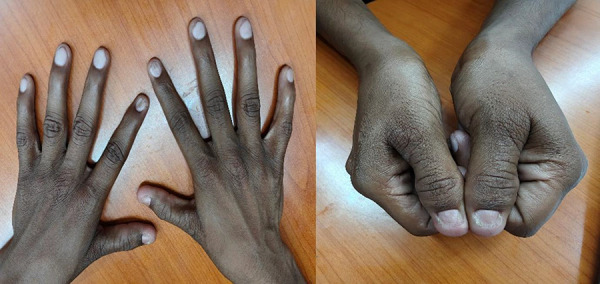
Dystrophic thumbnails and toenails.

**Table 1.** Investigations performed at the time of initial presentation.

**Laboratory parameter**	**Result**
Hemoglobin (g/dL)	13.5
Total WBC count (cells/µL)	5,600
Platelets (10^5^/µL)	2.5
Urine	Clear, yellow, pH = 5.0
Urine albumin / sugar	++++/nil
Urine pus cells/ RBCs	3/ nil
24 hours urine protein (g)	8.5
Urine PCR (mg/g)	10
Total Protein (g/dL)	5.8
Serum Albumin (g/dL)	2.8
Serum Creatinine (mg/dL)	1.1
Serum Urea (mg/dL)	22
Serum electrolytes (Na/K/Cl/HCO_3_) (mEq/L)	136/4.0/96/24
Total bilirubin	0.8 mg/dL
AST/ALT	20/18
Total cholesterol / LDL / HDL / TGL (mg/dL)	280 /140 /20/322

WBC = white blood cells; RBC = red blood cells; LDL = low density lipoprotein; HDL = high density lipoprotein; TGL = triglycerides.

**Table 3:** LMX1B (nail-patella syndrome) mutations with renal involvement.

**Nucleotide change**	**Protein change**	**Renal manifestation (clinical)**	**Type of renal involvement (proteinuria/course/histology)**	**Key extrarenal features**	**Reference**
c.791A>C	p.Gln264Pro	Nephrotic syndrome	Nephrotic-range proteinuria; biopsy FSGS; podocyte foot process effacement; progressive proteinuria	Nail dysplasia, no patellar hypoplasia	Present case
c.737G>A	p.Arg246Gln (R246Q)	Isolated hereditary FSGS	Nephrotic-range albuminuria; FSGS (NOS) on biopsy; several patients progressed to CKD/ESKD	No skeletal / nail features (isolated nephropathy)	Boyer et al. [11]
c.737G>C	p.Arg246Pro (R246P)	Minimal change nephrotic syndrome	Nephrotic syndrome; minimal change disease on biopsy; preserved eGFR	No skeletal / nail features (isolated nephropathy)	Boyer et al. [11]
c.819+1G>A	Splice-site mutation	Steroid-resistant nephrotic syndrome	Nephrotic-range proteinuria; FSGS on biopsy; diffuse foot process effacement; steroid resistant and progressive	Classic NPS (nail dysplasia, skeletal anomalies)	Nakata et al. [12]
c.709T>C	p.Ser237Pro	Early-onset ESRD with nephrotic syndrome	Heavy nephrotic-range proteinuria; FSGS on biopsy; severe podocyte injury; progression to ESRD in childhood	Dystrophic nails (NPS)	Carinelli et al. [13]
c.737G>T	p.Arg246Leu (R246L)	Isolated nephropathy in large family	Microscopic hematuria and/or proteinuria; glomerular disease; one member with ESRD on hemodialysis; others with preserved function	No skeletal / nail changes (nail-patella–like renal disease)	Liu et al. [14]
c.655C>G	p.Pro219Ala	FSGS with Alport-like phenotype	Proteinuria with mild renal dysfunction; FSGS on biopsy; GBM thinning and lamellation on EM	Sensorineural hearing loss (Alport-like)	Guan et al. [15]
c.725T>C	p.Val242Ala	NPS nephropathy with proteinuria	Proteinuria (variable); FSGS-type nephropathy; impaired DNA-binding in functional assays	Classic NPS (nails, patellae, skeletal)	Case with homeodomain mutation and renal disease reported in LMX1B literature summary and cohort analyses [1]
c.755A>G	p.Tyr252Cys	Familial ESKD	Progressive proteinuria leading to ESKD; FSGS on renal biopsy	Often minimal or absent skeletal features (isolated renal disease reported in FSGS cohorts)	[1]
c.773A>G	p.Asp258Gly	NPS nephropathy with GBM abnormalities	Proteinuria with glomerular disease; FSGS; GBM structural abnormalities on EM	NPS with ocular involvement (glaucoma reported in series)	[1]
c.845G>A	p.Arg282Gln	NPS with proteinuria	Persistent proteinuria; characteristic NPS nephropathy on biopsy	Classic NPS features	NPS kidney series summarizing LMX1B-associated proteinuric nephropathy [16]
IVS5+5G>A	Splice-site variant	NPS nephropathy	Proteinuria with FSGS-pattern glomerulopathy; GBM abnormalities typical of NPS	Classic NPS features	[1]
Multiple LMX1B variants (homeodomain-enriched)	Various	“NPS nephropathy” (2 – 62% of NPS cases)	Spectrum from asymptomatic proteinuria to nephrotic syndrome; FSGS most frequent; EM: irregular GBM thickening, electron-lucent zones, type III collagen fibrils; ~ 15% progress to ESKD	Iliac horns, patellar hypoplasia/aplasia, nail dysplasia, elbow dysplasia, glaucoma	Kidney disease in NPS: Lemley et al. [16]

FSGS = focal segmental glomerulosclerosis; NOS = not otherwise specified; CKD = chronic kidney disease; ESKD = end-stage kidney disease; eGFR = estimated glomerular filtration rate; NPS = nail-patella syndrome; GBM = glomerular basement membrane; EM = electron microscopy.

**Figure 1C:**
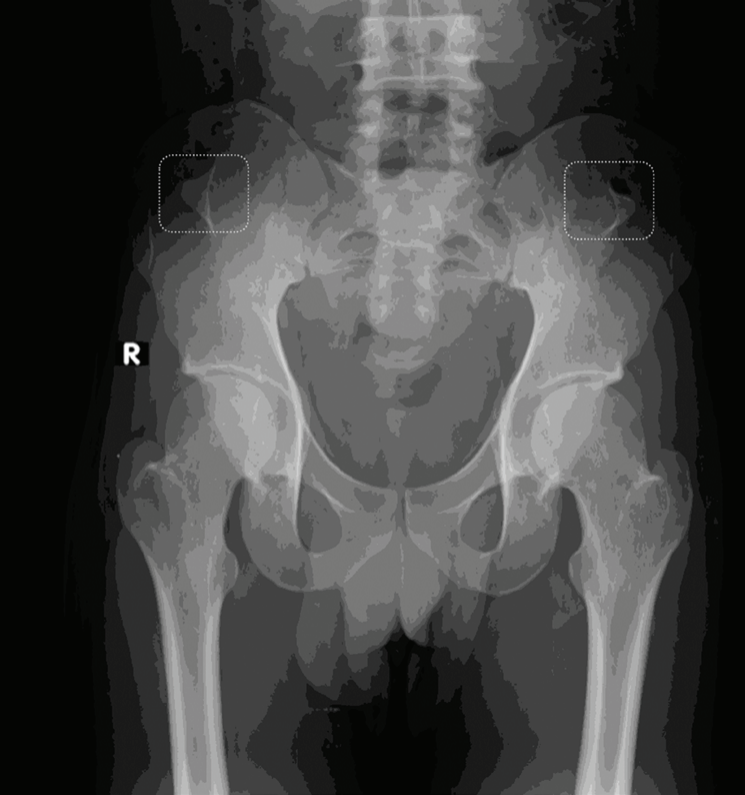
Pelvic X-ray showed bilateral iliac horns.

**Figure 1D:**
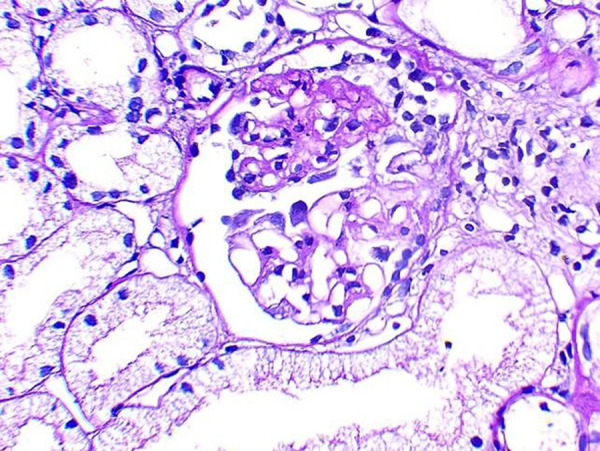
Renal biopsy revealed focal segmental glomerulosclerosis (NOS variant) with 20% interstitial fibrosis.

**Table 2:** Clinical manifestations of nail patella syndrome.

**System**		**Abnormality**
Musculoskeletal	Nails (98%)	Hypoplasia Dystrophic changes (abnormal ridging, splitting and triangular lunulae)
	Distal digits (> 90%)	Loss of creases in skin overlying distal interphalangeal joints Flexion and hyperextension abnormalities of PIPs, DIPs (swan neck deformity)
	Patella (75%)	Aplasia (20%) Hypoplasia Recurrent knee dislocation/subluxation
	Elbow (> 90%)	Posterior subluxation of radial head, Hypoplasia of lateral epicondyle and capitellum Cubitus valgus, Pterygia (webbing of the elbow)
	Pelvis (70%)	Iliac horns
Renal (30 – 40%)		Proteinuria from focal segmental glomerulosclerosis Progressive chronic kidney disease
Ophthalmological abnormalities (5%)		Open angle glaucoma Ocular hypertension Lester sign – hyperpigmented, irregular ring in the iris
Ears		Sensorineural hearing loss

PIP = proximal interphalengeal joint; DIP = distal interphalengeal joint.
